# Microbial Air Contamination in a Dental Setting Environment and Ultrasonic Scaling in Periodontally Healthy Subjects: An Observational Study

**DOI:** 10.3390/ijerph20032710

**Published:** 2023-02-03

**Authors:** Giovanni Boccia, Federica Di Spirito, Francesco D’Ambrosio, Francesco De Caro, Domenico Pecora, Riccardo Giorgio, Luigi Fortino, Walter Longanella, Gianluigi Franci, Biagio Santella, Massimo Amato

**Affiliations:** 1 Dai Dipartimento Di Igiene Sanitaria e Medicina Valutativa U.O.C. Igiene Ospedaliera, A.O.U. San Giovanni di Dio e Ruggi D’Aragona Largo Città di Ippocrate, 84131 Salerno, Italy; 2 Department of Medicine, Surgery and Dentistry, University of Salerno, 84081 Salerno, Italy; 3 A.O.U. San Giovanni di Dio e Ruggi D’Aragona, 84131 Salerno, Italy

**Keywords:** air contamination, microbial contamination, aerosol, dental setting, dental office, dental procedure, scaling, periodontal treatment

## Abstract

The risk of microbial air contamination in a dental setting, especially during aerosol-generating dental procedures (AGDPs), has long been recognized, becoming even more relevant during the COVID-19 pandemic. However, individual pathogens were rarely studied, and microbial loads were measured heterogeneously, often using low-sensitivity methods. Therefore, the present study aimed to assess microbial air contamination in the dental environment, identify the microorganisms involved, and determine their count by active air sampling at the beginning (T0), during (T1), and at the end (T2) of ultrasonic scaling in systemically and periodontally healthy subjects. Air microbial contamination was detected at T0 in all samples, regardless of whether the sample was collected from patients treated first or later; predominantly Gram-positive bacteria, including *Staphylococcus* and *Bacillus* spp. and a minority of fungi, were identified. The number of bacterial colonies at T1 was higher, although the species found were similar to that found during the T0 sampling, whereby Gram-positive bacteria, mainly *Streptococcus* spp., were identified. Air samples collected at T2 showed a decrease in bacterial load compared to the previous sampling. Further research should investigate the levels and patterns of the microbial contamination of air, people, and the environment in dental settings via ultrasonic scaling and other AGDPs and identify the microorganisms involved to perform the procedure- and patient-related risk assessment and provide appropriate recommendations for aerosol infection control.

## 1. Introduction

The human oral cavity hosts millions of microorganisms that colonize or infect the oral cavity and respiratory tract, such as bacteria, fungi, and viruses [1]. Thus, any dental procedure in which oral and respiratory fluids may be aerosolized will result in airborne contamination from these organisms, which in turn will result in the contamination of dental instruments and the entire environment, including oral healthcare workers and dental patients [2,3,4,5,6].

In detail, Micik et al. distinguished, based on their dimension, those inspirable particles produced by humans and the environment in a dental setting [7] into splatter, constituted by airborne particles greater than 50 microns in diameter and aerosols composed of particles less than 50 microns in diameter [8,9,10,11,12].

A visible aerosol cloud of particles and fluids [2] that originate from saliva, plaque, blood, calculus, and instrument water sources [13,14] and survive in the environment for a long time [15,16,17] is observed when employing an air–water syringe, dental handpieces, or ultrasonic scalers under irrigation to prevent teeth overheating [6,18]. Such procedures generating microbial aerosols or droplets less than 50 microns in diameter have been accordingly defined as aerosol-generating dental procedures (AGDPs) [19].

Moreover, a major concern during AGDPs is the spread of potentially pathogenic microorganisms through aerosol products, which pose a potential risk of transmitting infectious pathogens to oral healthcare providers, staff, patients, and visitors in the dental setting environment [2,3].

Indeed, the aerosol particles can carry potentially pathogenic bacteria such as *Staphylococcus* spp., *Streptococcus* spp., and *Mycobacterium tuberculosis*. Those bacteria can be transmitted and represent a potential mechanism for spreading infections among both oral healthcare workers and patients [2], especially considering that infected aerosols can remain airborne for minutes to hours [20,21], necessitating time for air exchange to eliminate this risk [22].

Periodontal treatment is routinely performed in primary dental care and is one of the most common treatments in daily dental practice, estimated to account for 44.5% of dental treatments in the adult population [23]. Active nonsurgical periodontal treatment aimed at the removal of supragingival and subgingival plaque and calculus [24] includes the use of ultrasonic and sonic scalers that reach a vibration frequency of 25,000–42,000 Hz [25], mechanical handpieces that apply prophylactic paste and rubber cups, handpieces that mix compressed air with abrasive powder, and hand instruments [26].

While more clarity is needed for the other biofilm removal methods, ultrasonic scaling is specifically included in the AGDP definitions. Indeed, during ultrasonic scaling, the aerosol visible to the naked eye is produced by interactions with the coolant and ultrasonic vibrations [24] and contains blood [27], bacteria, fungi, and likely viruses transported as droplets and splatter and traveling up to 3 m from the source [28].

Because of the recent COVID-19 pandemic and the fears and anxiety among dentists about the routes of transmission of SARS-CoV-2 and the associated risk of cross-infection [15], much attention has been given to the risk of airborne transmission and control measures. Most attention has been paid to the distance of aerosol and airborne contamination that occurs during dental procedures based on studies of the spread of aerosol/splatter contamination to individuals and the environment [29,30,31,32,33,34,35], while individual pathogens and microbial loads have been infrequently and inadequately studied. Therefore, the aim of the present observational study was to assess microbial air contamination in the dental environment with active air sampling to identify the microorganisms involved and to determine their count at the beginning, during, and end of ultrasonic scaling in periodontally healthy subjects.

## 2. Materials and Methods

### 2.1. Study Design and Sample

The present monocentric observational study, approved by the Local Ethics Committee, was performed in the Complex Operating Unit of Odontostomatology, Clinical Department of Head and Neck, Azienda Ospedaliero-Universitaria San Giovanni di Dio e Ruggi d’Aragona, Salerno, Italy, in accordance with the Code of Ethics of the World Medical Association (Declaration of Helsinki), between May and September 2022.

Air samples were collected randomly as part of the hospital’s standard microbiological air monitoring, which was performed once a week. At each random microbiological air monitoring session, at least two participants were consecutively enrolled among systemically and periodontally healthy outpatients scheduled for routine ultrasonic dental scaling.

The inclusion criteria were no smoking habits [36]; age ≥ 18 and ≤40 years [37]; ≥24 natural teeth; no clinical and radiological signs of active or untreated carious lesions, dental abscesses, gingivitis, and periodontitis; no fixed/removable prostheses, orthodontic appliances, and occlusal splints [38]; no lesions or normal variations of the oral mucosa [39]; and apparent good health.

The exclusion criteria were smoking habits (previous or current) [40]; age < 18 and >40 years [37]; <24 teeth; clinical and radiological evidence of active or untreated carious lesions, dental abscesses, gingivitis, and periodontitis [41]; dental implants with fixed/removable prostheses and orthodontic appliances and occlusal splints [38]; reactive, traumatic, disimmune, preneoplastic lesions or normal variations of the oral mucosa; oral and systemic infections; other comorbidities, drugs, and oral pathologies potentially affecting the oral, dental, and periodontal microbiome; neoplastic disease; medication-related osteonecrosis of the jaws [42,43]; pregnancy or lactation; corticosteroid or antibiotic administration in the past 3 months; and use of mouthwashes containing antiseptics or natural products with antimicrobial properties in the past 4 weeks [44].

The sample size was obtained from a previous study [45]. All enrolled subjects who voluntarily agreed to participate in the study had previously undergone oral and periodontal examination, panoramic radiography, and medical data collection and had given informed consent.

### 2.2. Ultrasonic Scaling Procedures

Mouth rinsing with chlorhexidine 0.20% was performed in all patients immediately before the procedure [46,47,48].

Supragingival scaling was performed by a single expert operator, equipped with a disposable gown and cap, face mask, and face shield, using the Stern Weber sc-a2 ultrasonic dental scaler with an independent source of distilled water and a sterile standard tip under a 150 L/min flow rate suction with a disposable saliva ejector with a diameter of 6.5 mm [49] placed in the corner of the mouth opposite of the quadrant to be treated.

The tip was kept in contact with the teeth as much as possible during the procedure and was cooled with a fine water spray (moderate setting) to minimize aerosol generation, although the high-volume evacuation system [50] was not used because of the absence of dental assistants. No polishing procedure was performed. The duration of each treatment was approximately 30–45 min.

### 2.3. Operating Area Disinfection and Cleaning Procedures

As previously proposed, the natural ventilation of the operating areas for at least 10–15 min was implemented between each patient. All operating room areas, from the least critical to the most critical, were adequately cleaned and disinfected [51].

The responsible personnel, wearing the appropriate PPE (at least gloves, surgical mask, cap, and goggles), performed the disinfection procedure consisting of the following steps:

- Cleaning and disinfecting all operating room surfaces;
- Cleaning the surfaces and handles of the furniture;
- Disinfecting the dental chair in the open and closed positions, especially the equipment that cannot be sterilized or the most frequently touched parts.

All surfaces were cleaned and disinfected with tuberculocidal, bactericidal, virucidal, and fungicidal agents containing benzalkonium chloride, phenyl phenol, isothiazolinone chloride, isopropyl butyl alcohols, and surfactants (Sporigerm, by IDS Spa, Savona 17100, Italy). The agents were used neatly and were allowed to act for at least 5 min according to the manufacturer’s recommendations, and then they were wiped with paper towels.

### 2.4. Air Samples Collection

Before sampling an environment, an appropriately trained person conducted an inspection to identify the sampling locations and delineate the area to be sampled, following an inspection plan previously developed based on the hazard analysis to verify:

- the availability and adequacy of the materials and equipment necessary for the collection, preparation, and shipment of samples;
- the verification of the sterile buffer solution used to collect and ship the samples for the absence of turbidity, flocculation, debris, or other foreign matter;
- the availability of the laboratory to receive and process the samples on schedule (within a maximum of 24 h after sample collection, keeping them refrigerated).

The sampling personnel carefully washed their hands and forearms and wore personal protective equipment such as gowns, masks, and gloves to avoid the contamination of the collected samples.

**Figure 1:**
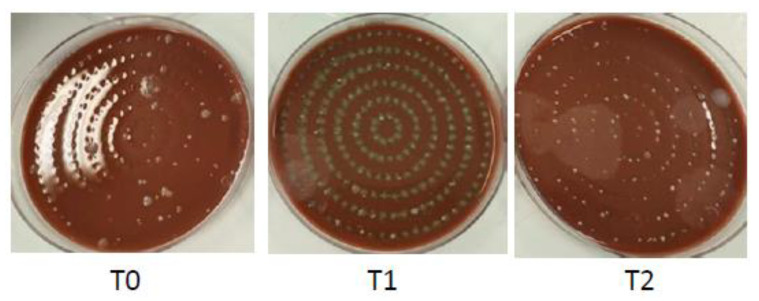
Chocolate Agar plates after T0, T1, and T2 air samplings.

Air samples were collected before using the SAS (Surface Air Sampler-SAS Super ISO USB, VWR^®^, Phillipsburg, NJ, USA). This bioaerosol sampler consists of an inlet cone, a 1 mm diameter 219-hole impactor stage, and an aluminum head and adapter for a standard Ø 90 mm Petri dish equipped with a Chocolate Agar (Becton Dickinson Chocolate Agar, GC II Agar with IsoVitaleX, Heidelberg, Germany), a universal medium for collecting most bacterial species. Air samples were collected for five minutes at an airflow rate of 180 L/min (Figure 1).

**Figure 2:**
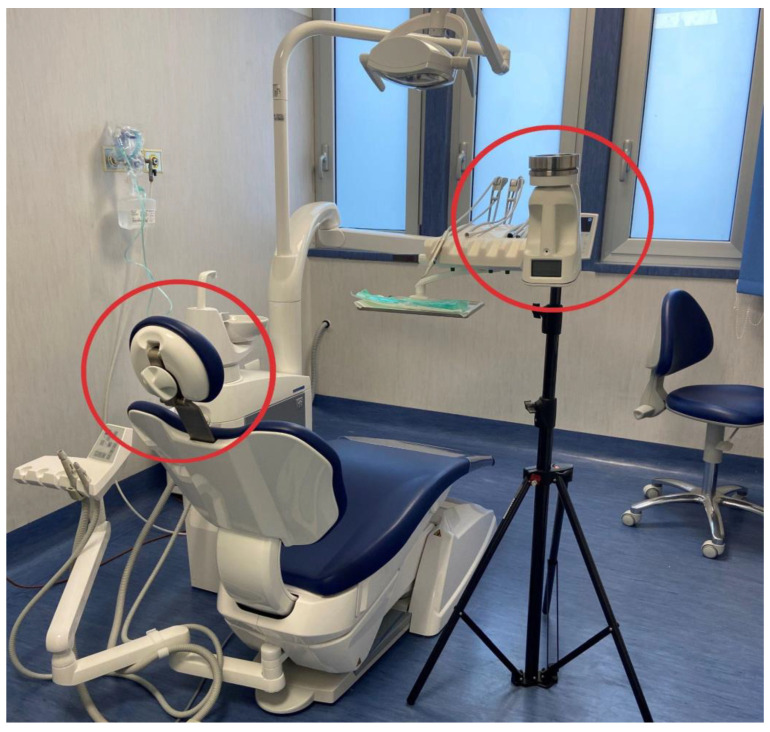
Air sampler position.

For each procedure, the air sampler was placed at the average working distance of the clinicians involved in the study (and from the aerosol source), 30 cm, at the height of 1.5 m above the floor, which corresponded to the area where the patient was breathing (Figure 2).

The samples were collected 5 min before starting the procedure (T0), during (T1), and immediately after (T2) the ultrasonic scaling. The agar plates were incubated at 36 ± 2 °C for 24–72 h, and colonies were counted. Colony counts were converted to concentrations in air (colony-forming units/cubic meter of air or CFU/m^3^)

Microbial identification was performed using a Matrix-Assisted Laser Desorption Ionization Time of Flight mass spectrometer (MALDI-TOF-MS, VITEK^®^ MS PRIME, bioMérieux Diagnostics, Bagno a Ripoli, Italy).

Those bacteria from the periodontal microbiome that require special media or growth conditions not currently used, such as mycobacteria or strict anaerobes, were not analyzed. Similarly, no viral particles such as influenza, *Rhinoviruses*, and SARS-*Coronavirus* were measured.

### 2.5. Colony-Forming Unit Assessment

The number of microorganisms counted on the surface of the plate was corrected for the statistical possibility of multiple particles passing through the same hole. The statistical formula was taken from the work of J. Maker [52]. The correction tables are given for the 90 mm Petri dish. The probable number was then used to calculate the CFU count per cubic meter of air sampled.

Finally, the mean value of the colony count was calculated for each sampling performed.

### 2.6. Statistical Analysis

Statistical analyses were performed using SPSS portable statistics version 19. Because of the normal distribution of the data, their mean was used as the statistical descriptor. The results are expressed as mean values, and intervals of CFU were calculated.

A Kruskal–Wallis test was performed to analyze the differences in microbial counts among the three sampling times in the same conditions.

Statistical significance was assumed at *p*-values less than 0.001.

## 3. Results

A total of 117 samples were collected by active air sampling during 39 ultrasonic scaling procedures from a group of 39 periodontally healthy subjects compliant with the eligibility criteria, 59% of whom were male and 41% were female.

No differences in identified microorganisms and colony counts were found between males and females at any point (before starting, during, and immediately after the ultrasonic scaling).

### 3.1. Air Samples Collected before Starting the Ultrasonic Scaling (T0)

When the samples were analyzed at time 0, before the start of the dental procedure, a very low number of bacterial colonies was detected. The same result was observed for each patient at time 0, regardless of whether the sample was collected from the patients treated first or later.

The species identified were mainly Gram-positive, including *Staphylococcus* spp. (40–60 CFU/m^3^), *Bacillus* spp., and fungi (5–15 CFU/m^3^).

### 3.2. Air Samples Collected during the Ultrasonic Scaling (T1)

The number of bacterial colonies in the samples collected at time 1, during ultrasonic scaling, was higher than in the previous sampling. Similar to the T0 sampling, the bacteria identified were mainly of the Gram-positive species and included *Staphylococcus* spp. And *Bacillus* spp., but with a greater number of colonies than the samples collected at time 0, 100–130 CFU/m^3^, and 10–20 CFU/m^3^.

The most abundant species belonged to the *Streptococcus* spp. family with a load of 200–300 CFU/m^3^, including *Streptococcus salivarius* and *Streptococcus mutans,* among the main species in the oral cavity.

### 3.3. Air Samples Collected Immediately after the Ultrasonic Scaling (T2)

The air samples collected immediately after ultrasonic scaling (T2) showed a decrease in bacterial load compared to the previous sampling, especially *Streptococcus* spp., which was reduced to 5–10 CFU/m^3^. *Staphylococcus* spp. was the main isolated species with a higher load (60–90 CFU/m^3^) than T1.

The identified microorganisms and mean values of the colony count calculated for each air sample collected before, during, and after ultrasonic scaling are synthesized in Table 1 and Supplementary Figure S1, which contains the T0 vs. T1 vs. T2 statistics with the alpha risk correction.

## 4. Discussion

The risk of microbial air contamination in the dental setting has long been recognized, becoming even more relevant during the COVID-19 pandemic [24,53,54,55,56]. However, individual pathogens were rarely studied, and microbial loads were measured heterogeneously, often using low-sensitivity methods [28]. Therefore, the present study aimed to assess microbial air contamination in the dental environment, identify the microorganisms involved, and determine their count by active air sampling at the beginning, during, and end of ultrasonic scaling in periodontally healthy subjects.

Previous studies investigating the spread of aerosol and splatter contamination to individuals and the environment during and after AGDPs [29,30,31,32,33,34,35] showed that the contamination rates were highest in the area around the dental unit. Such an area, likely penetrated by aerosols and splatter during AGDPs, was thus defined as the “red zone” and is recommended to be treated with careful cleaning and disinfection with antimicrobial agents after such procedures [57]. As expected, in the “red zone”, the patient and oral healthcare workers were most susceptible to contamination during AGDPs, including ultrasonic scaling [28]. Patient contamination was most common in the chest and facial areas, similar to dentists, and also depended on the working position [28]. Accordingly, in the present study, the air sampler was positioned 30 cm from the patient to simulate the dentist’s standard distance.

The near-operator position of the air sampler was also based on earlier findings describing the further circumferential diffusion of aerosol scatter generated during AGDPs [2,58] and is estimated to be up to about 1 m during crown preparation with a high-speed handpiece [59], but about 30 cm during ultrasonic scaling [4]; accordingly, distant passive gravimetric settlement air samples were not collected.

The reported differences in aerosol diffusion are likely due to the greater aerosolization associated with the higher rotational speed of the drills on high-speed handpieces compared to the vibration frequency of the tips on ultrasonic scalers [60].

In order to generalize the results, the vibration frequency of the tips of ultrasonic scalers and the source of cooling water were set as moderate in all procedures.

In addition, sonic scalers were not used, and polishing procedures were not performed as they are not currently listed in the AGDPs [19,61].

The low-volume evacuators presently employed were found to be roughly comparable to high-volume evacuators in terms of reducing contamination [62], although ledges are recommended in assisted AGDPs [63].

Different microbial species were identified in the air samples collected at different times during ultrasonic scaling.

In detail, the microbial species isolated before and immediately after the procedure were *Staphylococcus* spp., including *Staphylococcus hominis* and *Staphylococcus mutans*, which are commonly found in the environment. Similar species were reported by Read et al. [64], examining airborne microbial contamination on various surfaces in the dental setting, the area in front of the dental chair, and the dental chair itself. Additionally, the other bacteria isolated were mainly environmental microorganisms, such as *Micrococcus luteus*, *Staphylococcus haemolyticus*, *Staphylococcus lugdunensis*, and *Bacillus* ssp. The healthy periodontal conditions and the absence of mucosal and dental infections in the subjects studied could partially explain these results. Air samples similarly collected from periodontal subjects or those with oral, dental, or systemic infections might yield a more complex spectrum of microorganisms.

Conversely, the microbial air load differed significantly before starting and after AGDP in all subjects, with a proportional increase in commensal oral bacterial species, such as *Streptococcus salivarius* and *Streptococcus mutans*. These results could indicate a possible transmission of bacterial species from the patient to the practitioner and staff during ultrasonic scaling, even in healthy patients. Potentially pathogenic bacterial species were detected in this study; among them, Viridans group streptococci (VGS), especially *Streptococcus mitis* and *Streptococcus mutans,* were found to be increased during the ultrasonic scaling procedures, indicating a high risk of exposure to these pathogens, associated with the development of dental caries, gingivitis, and periodontitis. In addition, oral streptococci frequently have access to the bloodstream through periodontal lesions or oral abrasions formed from routine activities. This can lead to serious illnesses, including infective endocarditis and bacteremia. However, as has been shown for chlorhexidine-containing coolants that may reduce CFU [65], the present results may partially underestimate the actual oral microbial load of the aerosol during ultrasonic scaling due to preoperative antiseptic mouth rinsing [48,66,67,68], which is not only a common practice but still mandatory or strongly recommended during the COVID-19 epidemic [62,69].

The air samples collected after ultrasonic scaling (T2) showed the persistence of these bacterial species originating from the patient’s oral cavity, which were already detectable before starting the AGDP (T0), albeit in lower amounts.

Of note, microbial contamination of the air was detected even before ultrasonic scaling (T0), in accordance with Grenier et al.’s results [70], underscoring the risk of cross-infection in the dental environment not only associated with AGDPs but also with close contact during all dental procedures.

In addition, no differences were observed between individual patients in the preoperative (T0) samples, regardless of whether the sample was collected from patients treated first or later. This result contrasts with evidence that aerosols persist in room air for approximately 10–30 min after the end of AGDP [21], potentially increasing the risk of the transmission of microorganisms not only to oral health care personnel but also to subsequent patients, and is probably ascribable to the aeration, cleaning, and disinfection procedures presently performed [69].

Indeed, maintaining adequate cleanliness and ventilating the operating room after each patient is critical, as bacterial contamination in the air is reduced after adequate ventilation. Using personal protective equipment such as surgical masks, FFP2 masks, gloves, face shields, or eye protection is especially relevant for operators closest to the patient as oral healthcare workers [71,72]. Personal protective equipment crucially reduces the risk of infection transmission through droplet splashes from ultrasonic scalers and should be worn for some time after the procedure is completed [24].

Air cleaning systems [73] and suction systems with HEPA filters (high-efficiency particulate air), allowing generated aerosols to be conveyed outside the dental office, have also been proposed. Although expensive, these high-volume external suction systems reduce aerosols and splashes by up to 93–96% by extracting air at 100 cubic feet per minute [58,74,75].

However, evidence-based recommendations for mitigating infected aerosols in the dental setting should be continually re-evaluated according to the constant flow of new evidence and should be adapted to emerging and re-emerging infectious diseases that potentially impact oral and dental care and practice [76,77,78].

Despite the absence of a face mask sampling and distant passive gravimetric air sampling, even temporally after the completion of the procedure, and despite the inability to identify anaerobic bacteria and viruses and the preoperative antiseptic mouth rinse potentially altering the microbial load and species identified, the present study may be the first evaluating microbial air contamination by active air sampling and identifying the microorganisms involved and their numbers in periodontally healthy subjects under controlled operative conditions. In addition, the present study assessed and pointed out microbial air contamination even before ultrasonic scaling.

Future studies should combine various active and passive air sampling methods to evaluate microbial air contamination before, during, immediately after, and some time after the completion of ultrasonic scaling and other AGDPs to accurately assess the procedure-related risk of aerosol transmission in the dental setting and provide appropriate infection control measures accordingly.

In addition, further studies should integrate data on microbial air contamination from ultrasonic scaling and other AGDPs [66] with microbiologically examined gingival crevicular and saliva samples and oropharyngeal swabs from systemically and periodontally healthy young and adult subjects and periodontal patients to identify correspondent transmissible pathogens from the oral cavity or other body sites and perform the appropriate patient-related risk assessment for airborne and aerosol-transmitted infections in a dental setting. A patient-related risk assessment may be particularly important to reduce the potential spread of oral, dental, and periodontal microorganisms in immunocompromised patients and to prevent healthcare-associated infections and emerging and reemerging infections [48,77].

## 5. Conclusions

Air microbial contamination was detected before ultrasonic scaling (T0) in all samples from systemically and periodontally healthy patients, regardless of whether the sample was collected from patients treated first or later. Predominantly Gram-positive bacteria, including *Staphylococcus* spp. and *Bacillus* spp., and a minority of fungi were identified.

The number of bacterial colonies in the samples collected during ultrasonic scaling (T1) was higher, although, similar to the T0 sampling, the bacteria identified were predominantly of the Gram-positive species, but in this time the most abundant species belonged to the *Streptococcus* spp. family with a load of 200–300 CFU/m^3^, including *Streptococcus salivarius* and *Streptococcus mutans*. Air samples collected immediately after ultrasonic scaling (T2) showed a decrease in bacterial load compared to the previous sampling.

Further research using comparable and combined methodological approaches should investigate the levels and patterns of the microbial contamination of air, people, and the environment in a dental setting via ultrasonic scaling and other AGDPs and identify the microorganisms involved to perform the procedure- and patient-related risk assessment and provide appropriate recommendations for aerosol infection control.

Considering that during ultrasonic scaling and daily dental procedures, bacteria, viruses, blood, and fungi are present in the aerosol itself with a high probability, it is essential that this generated aerosol be appropriately controlled to prevent the infection of patients and oral healthcare workers.

## Figures and Tables

**Table 1:** Colony counts of identified microorganisms with median values and [relative IQR] from air samples collected before, during, and after ultrasonic scaling of periodontally healthy subjects.

Species	T 0	T 1	T 2	*p*-Value
*Staphylococcus* spp. (*S. capitis, S. haemolyticus,* *S. hominis*)	50.0 CFU/m^3^ [45.0, 58.0]	124.0 CFU/m^3^ [111.0, 140.0]	70.0 CFU/m^3^ [65.5, 78.0]	<0.001
Viridans streptococci (*S. mitis, S. salivarius,* *S. mutans*)	0 CFU/m^3^ [0, 0]	256.0 CFU/m^3^ [244.0, 288.0]	6.0 CFU/m^3^ [5.0, 8.0]	<0.001
Others (*Bacillus* spp. and fungi)	7.0 CFU/m^3^ [6.0, 8.0]	15.0 CFU/m^3^ [11.0, 17.0]	18.0 CFU/m^3^ [15.5, 19.0]	<0.001

## Data Availability

Data are available on PubMed/MEDLINE, Scopus, and Web of Science databases.

## Supplementary Materials

The following supporting information can be downloaded at https://www.mdpi.com/article/10.3390/ijerph20032710/s1: Figure S1: The data T0 vs. T1 vs. T2 were statistically analyzed using a posthoc test with the correction alpha using the Bonferroni method.
