# Supplementary material for: Microbial Air Contamination in a Dental Setting Environment and Ultrasonic Scaling in Periodontally Healthy Subjects: An Observational Study
Source: Int J Environ Res Public Health. 2023 Feb 3;20(3):2710. doi: 10.3390/ijerph20032710 (PMC9916071; doi:10.3390/ijerph20032710)
Supplement: Supplementary file 1 [file ijerph-20-02710-s001.zip › ijerph-2125524-supplementary.pdf]

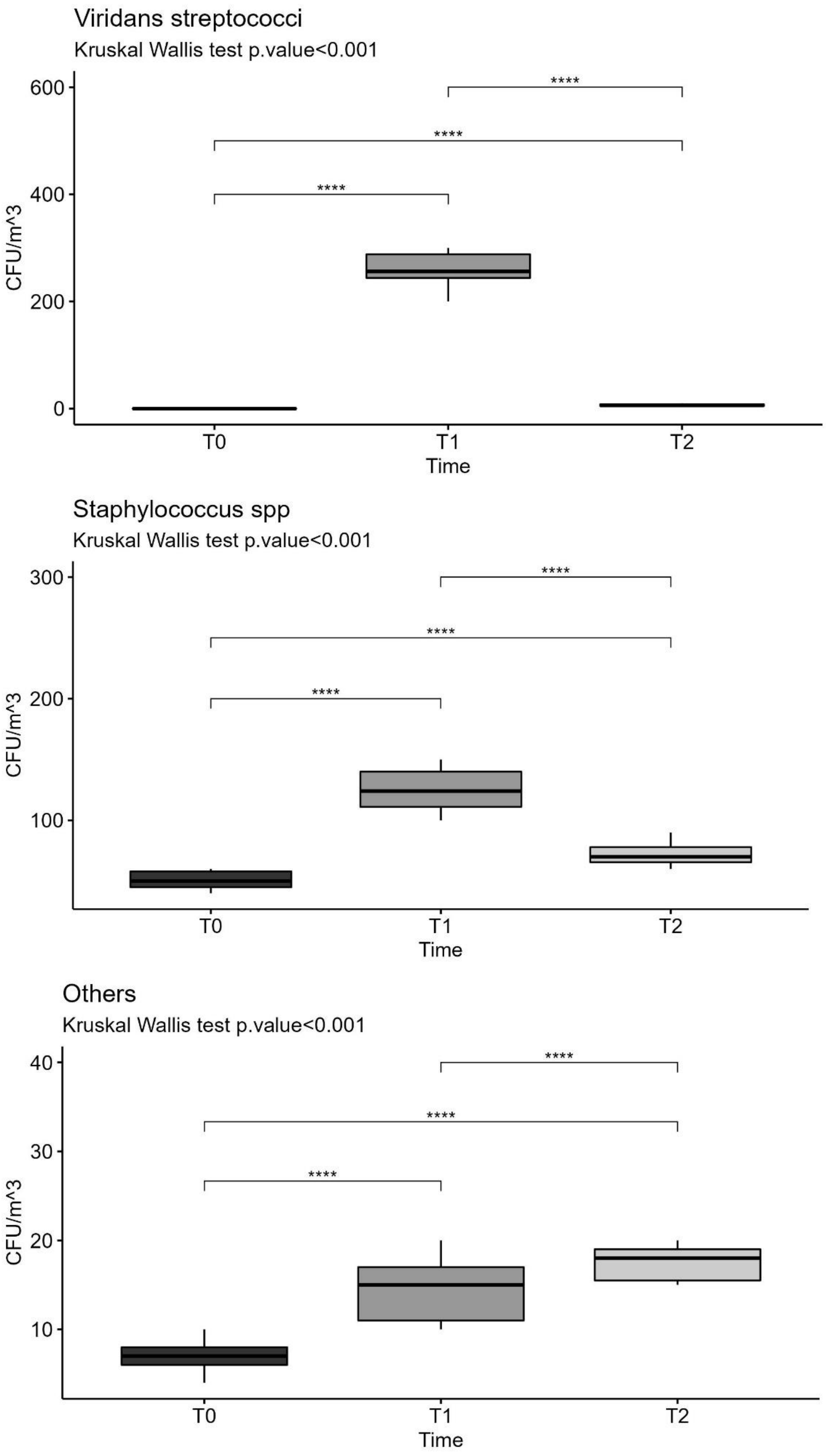

Supplementary Figure S1: The data T0 vs T1 vs T2 were statistically analyzed using a post hoc test with the correction alfa using the Bonferroni method.
